# Diisopropyl 3-[(*E*)-2-(3,4-dimethoxy­phen­yl)ethen­yl]-5-oxocyclo­hex-3-ene-1,1-dicarboxyl­ate

**DOI:** 10.1107/S160053680901544X

**Published:** 2009-05-07

**Authors:** Shifeng Chen, Chen Zhang, Jian-feng Qi

**Affiliations:** a Department of Medicinal Chemistry, College of Pharmaceutical Science, Zhejiang University, Hangzhou 310058, Zhejiang, People’s Republic of China

## Abstract

The title compound, C_24_H_30_O_7_, displays a *trans* configuration with respect to the C=C bond. The cyclo­hexenone ring has an envelope conformation; the flap atom (with the isopropoxycarbonyl groups) is displaced by 0.664 (3) Å from the plane of the other five ring atoms and the carbonyl O atom. The dihedral angle between the cyclo­hexenone ring and the benzene ring is 7.85 (9)°. The *meta* and *para* meth­oxy O atoms are displaced by 0.003 (7) and 0.031 (4) Å, respectively, from the benzene ring to which they are attached.

## Related literature

For the biological activities of cyclo­hex-2-enone derivatives, see: Correia *et al.* (2001 ▶); Stadler *et al.* (1994 ▶). Cyclo­hex-2-enone derivatives can be used as precursors in the synthesis of various compounds such as vitamin E (Hu *et al.*, 2003 ▶).
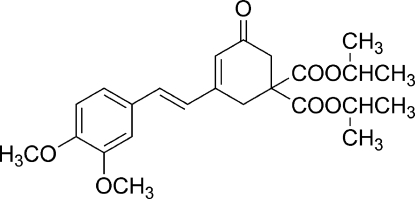


## Experimental

### 

#### Crystal data

C_24_H_30_O_7_
*M* *_r_* = 430.50
Monoclinic, 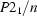

*a* = 8.9228 (6) Å
*b* = 13.3886 (7) Å
*c* = 20.2009 (11) Å
β = 104.2488 (14)°
*V* = 2339.0 (2) Å^3^
*Z* = 4
Mo *K*α radiation
μ = 0.09 mm^−1^
*T* = 296 K
0.38 × 0.30 × 0.06 mm

#### Data collection

Rigaku R-AXIS RAPID diffractometer
Absorption correction: multi-scan (*ABSCOR*; Higashi, 1995 ▶) *T*_min_ = 0.952, *T*_max_ = 0.995
22350 measured reflections
5332 independent reflections
2404 reflections with *F* ^2^ > 2σ(*F* ^2^)
*R*_int_ = 0.050

#### Refinement

*R*[*F* ^2^ > 2σ(*F* ^2^)] = 0.062
*wR*(*F* ^2^) = 0.169
*S* = 1.00
5332 reflections
282 parameters
H-atom parameters constrained
Δρ_max_ = 0.40 e Å^−3^
Δρ_min_ = −0.32 e Å^−3^

### 

Data collection: *PROCESS-AUTO* (Rigaku, 1998 ▶); cell refinement: *PROCESS-AUTO*; data reduction: *CrystalStructure* (Rigaku/MSC, 2004 ▶); program(s) used to solve structure: *SHELXS97* (Sheldrick, 2008 ▶); program(s) used to refine structure: *SHELXL97* (Sheldrick, 2008 ▶); molecular graphics: *ORTEP-3 for Windows* (Farrugia, 1997 ▶); software used to prepare material for publication: *CrystalStructure*.

## Supplementary Material

Crystal structure: contains datablocks global, I. DOI: 10.1107/S160053680901544X/kp2217sup1.cif

Structure factors: contains datablocks I. DOI: 10.1107/S160053680901544X/kp2217Isup2.hkl

Additional supplementary materials:  crystallographic information; 3D view; checkCIF report

## Figures and Tables

**Fig. 1.**
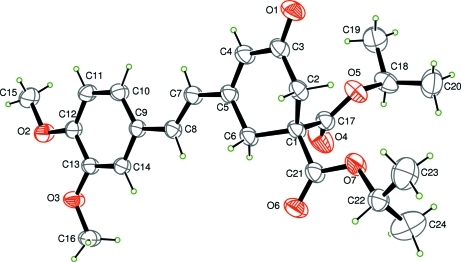
The molecular structure of (I) with atom labels showing the 50% probability displacement ellipsoids.

## supplementary crystallographic information

### Comment

Cyclohex-2-enone derivatives have significant biological activities such as anti-bacterial Stadler *et al.*, 1994), anti-cancer (Correia *et al.*, 2001). In addition, cyclohex-2-enone derivatives can be used as precursors in the synthesis of various compounds such as vitamin E (Hu *et al.*, 2003). We are interested in their pharmaceutical properties. In this paper, we present the X-ray crystal structure analysis of the title compound (I) (Fig. 1). The molecule displays a *trans* configuration with respect to the C═C. The cyclohexenone ring has an envelope conformation, the plane which is defined by the atoms C1, C2 and C6 (forming the flap) and the plane defined by C2, C3, C4, C5, and C6  form a dihedral angle of 48.95 (0)°. The dihedral angle between the cyclohexenone ring and the benzene ring is 7.85 (9)°. The *meta* and *para O*-methoxy atoms are displaced by 0.003 (7)Å and 0.031 (4)Å from the benzene ring to which they are attached. The carbon atom C7 of the C═C is displaced by 0.182 (6)Å from the C2, C3, C4, C5, C6 plane, whereas the carbon atom C8 is displaced by 0.011 (3)Å from the benzene ring.

### Experimental

A solution of (*R*,*S*)-methyl 3-methyl-5-oxo -1-phenylcyclohex-3-ene-1-carboxylate (0.5 mmol), Veratraldehyde (2 mmol), and piperidine (0.75 mmol) in 4 ml 2-propanol was heated at 373 K for 24 h. The reaction mixture was acidified with dilute aqueous HCl, concentrated, and partitioned between water and ethyl acetate. The pure product was obtained through silica gel chromatography, and diffraction quality crystals were obtained by slow evaporation of an ethyl acetate/petroleum ether/ dichloromethane (1:2:2) solution at room temperature.

### Refinement

All H atoms were placed in calculated positions, with C—H distances in the range 0.93–0.98Å and included in the final cycles of refinement in the riding-model approximation, with *U*_iso_(H) = 1.2*U*_eq_(C).

### Crystal data

**Table:**

C_24_H_30_O_7_	*F*(000) = 920.00
*M**_r_* = 430.50	*D*_x_ = 1.222 Mg m^−^^3^
Monoclinic, *P*2_1_/*n*	Mo *K*α radiation, λ = 0.71075 Å
Hall symbol: -P 2yn	Cell parameters from 11134 reflections
*a* = 8.9228 (6) Å	θ = 3.0–27.4°
*b* = 13.3886 (7) Å	µ = 0.09 mm^−^^1^
*c* = 20.2009 (11) Å	*T* = 296 K
β = 104.2488 (14)°	Platelet, yellow
*V* = 2339.0 (2) Å^3^	0.38 × 0.30 × 0.06 mm
*Z* = 4	

### Data collection

**Table:**

Rigaku R-AXIS RAPID diffractometer	2404 reflections with *F*^2^ > 2σ(*F*^2^)
Detector resolution: 10.00 pixels mm^-1^	*R*_int_ = 0.050
ω scans	θ_max_ = 27.4°
Absorption correction: multi-scan (*ABSCOR*; Higashi, 1995)	*h* = −11→11
*T*_min_ = 0.952, *T*_max_ = 0.995	*k* = −17→16
22350 measured reflections	*l* = −26→26
5332 independent reflections	

### Refinement

**Table:**

Refinement on *F*^2^	*w* = 1/[σ^2^(*F*_o_^2^) + (0.0371*P*)^2^ + 2*P*] where *P* = (*F*_o_^2^ + 2*F*_c_^2^)/3
*R*[*F*^2^ > 2σ(*F*^2^)] = 0.062	(Δ/σ)_max_ < 0.001
*wR*(*F*^2^) = 0.169	Δρ_max_ = 0.40 e Å^−^^3^
*S* = 1.00	Δρ_min_ = −0.32 e Å^−^^3^
5332 reflections	Extinction correction: *SHELXL97* (Sheldrick, 2008)
282 parameters	Extinction coefficient: 0.0087 (8)
H-atom parameters constrained	

### Special details

**Table:**

Geometry. ENTER SPECIAL DETAILS OF THE MOLECULAR GEOMETRY
Refinement. Refinement using reflections with *F*^2^ > 2.0 σ(*F*^2^). The weighted *R*-factor(*wR*), goodness of fit (*S*) and *R*-factor (gt) are based on *F*, with *F* set to zero for negative *F*. The threshold expression of *F*^2^ > 2.0 σ(*F*^2^) is used only for calculating *R*-factor (gt).

### Fractional atomic coordinates and isotropic or equivalent isotropic displacement parameters (Å^2^)

**Table:**

	*x*	*y*	*z*	*U*_iso_*/*U*_eq_	
O1	−0.0208 (2)	0.25454 (16)	0.26220 (12)	0.0861 (7)	
O2	1.1752 (2)	0.54715 (16)	0.43247 (12)	0.0769 (6)	
O3	1.0310 (2)	0.70068 (13)	0.37276 (12)	0.0775 (6)	
O4	0.1738 (3)	0.4751 (2)	0.10365 (13)	0.1100 (10)	
O5	−0.0202 (2)	0.37309 (14)	0.10638 (11)	0.0750 (6)	
O6	−0.0166 (2)	0.67279 (16)	0.18181 (13)	0.0957 (8)	
O7	−0.1964 (2)	0.56180 (14)	0.13391 (13)	0.0922 (8)	
C1	0.0411 (2)	0.49558 (19)	0.19360 (14)	0.0544 (7)	
C2	−0.0489 (3)	0.4253 (2)	0.22908 (14)	0.0599 (7)	
C3	0.0432 (3)	0.3344 (2)	0.25655 (16)	0.0622 (7)	
C4	0.2096 (3)	0.3458 (2)	0.27832 (16)	0.0652 (8)	
C5	0.2829 (3)	0.4331 (2)	0.27522 (14)	0.0558 (7)	
C6	0.1953 (2)	0.5246 (2)	0.24290 (14)	0.0586 (7)	
C7	0.4482 (3)	0.4387 (2)	0.30523 (14)	0.0597 (7)	
C8	0.5383 (2)	0.5190 (2)	0.31062 (13)	0.0544 (7)	
C9	0.7036 (2)	0.5223 (2)	0.34373 (13)	0.0519 (6)	
C10	0.7840 (3)	0.4414 (2)	0.37748 (14)	0.0618 (7)	
C11	0.9407 (3)	0.4469 (2)	0.40793 (16)	0.0639 (8)	
C12	1.0205 (2)	0.5343 (2)	0.40526 (14)	0.0579 (7)	
C13	0.9414 (3)	0.6176 (2)	0.37214 (14)	0.0559 (7)	
C14	0.7853 (2)	0.6114 (2)	0.34175 (13)	0.0548 (7)	
C15	1.2624 (3)	0.4625 (2)	0.46223 (19)	0.0837 (10)	
C16	0.9583 (3)	0.7883 (2)	0.3402 (2)	0.0840 (10)	
C17	0.0745 (3)	0.4477 (2)	0.12976 (16)	0.0606 (7)	
C18	−0.0112 (4)	0.3231 (2)	0.04279 (18)	0.0806 (10)	
C19	0.0580 (4)	0.2226 (2)	0.0621 (2)	0.1038 (13)	
C20	−0.1745 (5)	0.3175 (3)	−0.0010 (2)	0.1269 (17)	
C21	−0.0570 (3)	0.5888 (2)	0.16937 (16)	0.0640 (8)	
C22	−0.3203 (3)	0.6375 (2)	0.1150 (2)	0.0826 (10)	
C23	−0.4640 (4)	0.5853 (3)	0.1200 (2)	0.1157 (14)	
C24	−0.3221 (5)	0.6699 (3)	0.0460 (2)	0.1302 (17)	
H4	0.2689	0.2901	0.2953	0.078*	
H7	0.4966	0.3794	0.3226	0.072*	
H8	0.4922	0.5781	0.2917	0.065*	
H10	0.7316	0.3819	0.3798	0.074*	
H11	0.9923	0.3915	0.4303	0.077*	
H14	0.7334	0.6670	0.3197	0.066*	
H18	0.0539	0.3618	0.0195	0.097*	
H22	−0.2992	0.6941	0.1467	0.099*	
H61	0.2579	0.5607	0.2179	0.070*	
H62	0.1747	0.5671	0.2785	0.070*	
H151	1.2164	0.4340	0.4961	0.100*	
H152	1.3666	0.4826	0.4832	0.100*	
H153	1.2632	0.4138	0.4274	0.100*	
H161	0.9225	0.7765	0.2920	0.101*	
H162	1.0312	0.8424	0.3480	0.101*	
H163	0.8722	0.8052	0.3587	0.101*	
H191	0.1621	0.2302	0.0891	0.125*	
H192	−0.0021	0.1875	0.0880	0.125*	
H193	0.0585	0.1854	0.0215	0.125*	
H201	−0.2119	0.3837	−0.0138	0.152*	
H202	−0.2395	0.2861	0.0243	0.152*	
H203	−0.1760	0.2792	−0.0413	0.152*	
H221	−0.1428	0.4046	0.1965	0.072*	
H222	−0.0754	0.4606	0.2666	0.072*	
H231	−0.5474	0.6325	0.1133	0.139*	
H232	−0.4487	0.5553	0.1643	0.139*	
H233	−0.4888	0.5345	0.0855	0.139*	
H241	−0.3863	0.7280	0.0348	0.156*	
H242	−0.3624	0.6172	0.0144	0.156*	
H243	−0.2187	0.6857	0.0434	0.156*	

### Atomic displacement parameters (Å^2^)

**Table:**

	*U*^11^	*U*^22^	*U*^33^	*U*^12^	*U*^13^	*U*^23^
O1	0.0713 (15)	0.0646 (13)	0.1168 (19)	−0.0242 (11)	0.0124 (13)	0.0059 (13)
O2	0.0417 (11)	0.0712 (13)	0.1039 (16)	−0.0005 (10)	−0.0083 (10)	0.0168 (12)
O3	0.0498 (11)	0.0530 (11)	0.1163 (18)	−0.0075 (9)	−0.0049 (11)	0.0131 (11)
O4	0.104 (2)	0.131 (2)	0.113 (2)	−0.0563 (18)	0.0592 (17)	−0.0351 (17)
O5	0.0790 (15)	0.0680 (13)	0.0850 (15)	−0.0222 (11)	0.0339 (12)	−0.0240 (11)
O6	0.0630 (14)	0.0491 (12)	0.154 (2)	−0.0057 (10)	−0.0133 (14)	−0.0040 (13)
O7	0.0500 (12)	0.0593 (12)	0.142 (2)	0.0089 (10)	−0.0239 (13)	−0.0159 (13)
C1	0.0424 (14)	0.0469 (14)	0.0709 (18)	−0.0037 (12)	0.0080 (13)	−0.0053 (13)
C2	0.0481 (16)	0.0607 (17)	0.0698 (18)	−0.0081 (13)	0.0123 (14)	−0.0103 (15)
C3	0.0539 (17)	0.0587 (17)	0.0725 (19)	−0.0127 (14)	0.0128 (14)	−0.0055 (15)
C4	0.0499 (17)	0.0549 (16)	0.085 (2)	−0.0041 (13)	0.0064 (15)	0.0004 (15)
C5	0.0458 (15)	0.0519 (15)	0.0669 (17)	−0.0029 (12)	0.0081 (13)	−0.0029 (13)
C6	0.0438 (15)	0.0527 (15)	0.0743 (19)	−0.0078 (12)	0.0048 (13)	−0.0062 (14)
C7	0.0432 (15)	0.0552 (16)	0.0760 (19)	−0.0012 (13)	0.0057 (13)	0.0001 (14)
C8	0.0432 (14)	0.0535 (15)	0.0640 (17)	0.0005 (12)	0.0081 (12)	−0.0030 (13)
C9	0.0394 (14)	0.0523 (15)	0.0611 (16)	0.0004 (12)	0.0067 (12)	0.0000 (13)
C10	0.0475 (16)	0.0554 (16)	0.079 (2)	−0.0050 (13)	0.0078 (14)	0.0078 (15)
C11	0.0479 (16)	0.0578 (17)	0.079 (2)	0.0044 (14)	0.0020 (14)	0.0129 (15)
C12	0.0363 (14)	0.0605 (17)	0.0701 (18)	0.0011 (12)	0.0004 (12)	0.0070 (14)
C13	0.0439 (15)	0.0491 (15)	0.0701 (18)	−0.0023 (12)	0.0053 (13)	0.0025 (13)
C14	0.0415 (15)	0.0488 (15)	0.0687 (18)	0.0033 (11)	0.0034 (13)	0.0021 (13)
C15	0.0513 (18)	0.083 (2)	0.103 (2)	0.0133 (17)	−0.0069 (17)	0.016 (2)
C16	0.072 (2)	0.0537 (18)	0.117 (2)	−0.0051 (16)	0.006 (2)	0.0135 (18)
C17	0.0520 (17)	0.0572 (17)	0.0704 (19)	−0.0043 (14)	0.0109 (14)	−0.0038 (15)
C18	0.099 (2)	0.068 (2)	0.086 (2)	−0.0129 (19)	0.044 (2)	−0.0218 (18)
C19	0.093 (2)	0.094 (2)	0.121 (3)	0.021 (2)	0.020 (2)	−0.022 (2)
C20	0.137 (4)	0.120 (3)	0.098 (3)	0.025 (3)	−0.021 (2)	−0.023 (2)
C21	0.0441 (16)	0.0577 (17)	0.085 (2)	−0.0044 (13)	0.0054 (15)	−0.0073 (16)
C22	0.056 (2)	0.068 (2)	0.109 (2)	0.0014 (16)	−0.0084 (18)	−0.0013 (19)
C23	0.074 (2)	0.146 (4)	0.125 (3)	0.008 (2)	0.019 (2)	0.003 (3)
C24	0.134 (4)	0.135 (4)	0.142 (4)	0.045 (3)	0.072 (3)	0.031 (3)

### Geometric parameters (Å, °)

**Table:**

O1—C3	1.231 (3)	C22—C24	1.456 (6)
O2—C12	1.366 (3)	C2—H221	0.970
O2—C15	1.421 (3)	C2—H222	0.970
O3—C13	1.368 (3)	C4—H4	0.930
O3—C16	1.421 (3)	C6—H61	0.970
O4—C17	1.195 (4)	C6—H62	0.970
O5—C17	1.318 (3)	C7—H7	0.930
O5—C18	1.468 (4)	C8—H8	0.930
O6—C21	1.189 (3)	C10—H10	0.930
O7—C21	1.323 (3)	C11—H11	0.930
O7—C22	1.479 (3)	C14—H14	0.930
C1—C2	1.526 (4)	C15—H151	0.960
C1—C6	1.536 (3)	C15—H152	0.960
C1—C17	1.534 (4)	C15—H153	0.960
C1—C21	1.534 (3)	C16—H161	0.960
C2—C3	1.497 (3)	C16—H162	0.960
C3—C4	1.449 (4)	C16—H163	0.960
C4—C5	1.348 (4)	C18—H18	0.980
C5—C6	1.512 (3)	C19—H191	0.960
C5—C7	1.452 (3)	C19—H192	0.960
C7—C8	1.331 (3)	C19—H193	0.960
C8—C9	1.462 (3)	C20—H201	0.960
C9—C10	1.383 (3)	C20—H202	0.960
C9—C14	1.404 (3)	C20—H203	0.960
C10—C11	1.384 (3)	C22—H22	0.980
C11—C12	1.377 (4)	C23—H231	0.960
C12—C13	1.399 (3)	C23—H232	0.960
C13—C14	1.379 (3)	C23—H233	0.960
C18—C19	1.492 (5)	C24—H241	0.960
C18—C20	1.509 (5)	C24—H242	0.960
C22—C23	1.485 (5)	C24—H243	0.960
			
C12—O2—C15	117.6 (2)	C5—C6—H62	109.1
C13—O3—C16	117.9 (2)	H61—C6—H62	109.5
C17—O5—C18	119.4 (2)	C5—C7—H7	116.3
C21—O7—C22	119.7 (2)	C8—C7—H7	116.3
C2—C1—C6	110.0 (2)	C7—C8—H8	117.2
C2—C1—C17	111.8 (2)	C9—C8—H8	117.2
C2—C1—C21	109.0 (2)	C9—C10—H10	119.3
C6—C1—C17	108.8 (2)	C11—C10—H10	119.3
C6—C1—C21	110.6 (2)	C10—C11—H11	119.9
C17—C1—C21	106.6 (2)	C12—C11—H11	119.9
C1—C2—C3	111.8 (2)	C9—C14—H14	119.6
O1—C3—C2	121.0 (2)	C13—C14—H14	119.6
O1—C3—C4	121.9 (2)	O2—C15—H151	109.5
C2—C3—C4	117.0 (2)	O2—C15—H152	109.5
C3—C4—C5	123.4 (2)	O2—C15—H153	109.5
C4—C5—C6	121.1 (2)	H151—C15—H152	109.5
C4—C5—C7	118.7 (2)	H151—C15—H153	109.5
C6—C5—C7	120.2 (2)	H152—C15—H153	109.5
C1—C6—C5	111.1 (2)	O3—C16—H161	109.5
C5—C7—C8	127.3 (2)	O3—C16—H162	109.5
C7—C8—C9	125.6 (2)	O3—C16—H163	109.5
C8—C9—C10	123.0 (2)	H161—C16—H162	109.5
C8—C9—C14	119.0 (2)	H161—C16—H163	109.5
C10—C9—C14	118.0 (2)	H162—C16—H163	109.5
C9—C10—C11	121.4 (2)	O5—C18—H18	110.2
C10—C11—C12	120.2 (2)	C19—C18—H18	110.2
O2—C12—C11	124.9 (2)	C20—C18—H18	110.2
O2—C12—C13	115.7 (2)	C18—C19—H191	109.5
C11—C12—C13	119.4 (2)	C18—C19—H192	109.5
O3—C13—C12	114.8 (2)	C18—C19—H193	109.5
O3—C13—C14	125.2 (2)	H191—C19—H192	109.5
C12—C13—C14	120.0 (2)	H191—C19—H193	109.5
C9—C14—C13	120.9 (2)	H192—C19—H193	109.5
O4—C17—O5	123.6 (3)	C18—C20—H201	109.5
O4—C17—C1	124.1 (2)	C18—C20—H202	109.5
O5—C17—C1	112.2 (2)	C18—C20—H203	109.5
O5—C18—C19	106.9 (2)	H201—C20—H202	109.5
O5—C18—C20	106.4 (3)	H201—C20—H203	109.5
C19—C18—C20	112.7 (3)	H202—C20—H203	109.5
O6—C21—O7	124.7 (2)	O7—C22—H22	110.5
O6—C21—C1	125.6 (2)	C23—C22—H22	110.5
O7—C21—C1	109.7 (2)	C24—C22—H22	110.5
O7—C22—C23	105.2 (2)	C22—C23—H231	109.5
O7—C22—C24	106.4 (3)	C22—C23—H232	109.5
C23—C22—C24	113.5 (3)	C22—C23—H233	109.5
C1—C2—H221	108.9	H231—C23—H232	109.5
C1—C2—H222	108.9	H231—C23—H233	109.5
C3—C2—H221	108.9	H232—C23—H233	109.5
C3—C2—H222	108.9	C22—C24—H241	109.5
H221—C2—H222	109.5	C22—C24—H242	109.5
C3—C4—H4	118.3	C22—C24—H243	109.5
C5—C4—H4	118.3	H241—C24—H242	109.5
C1—C6—H61	109.1	H241—C24—H243	109.5
C1—C6—H62	109.1	H242—C24—H243	109.5
C5—C6—H61	109.1		
			
C15—O2—C12—C11	4.0 (4)	C21—C1—C17—O4	81.0 (3)
C15—O2—C12—C13	−175.5 (2)	C21—C1—C17—O5	−98.3 (2)
C16—O3—C13—C12	−179.8 (2)	C1—C2—C3—O1	−151.3 (3)
C16—O3—C13—C14	0.7 (4)	C1—C2—C3—C4	30.9 (3)
C17—O5—C18—C19	108.2 (3)	O1—C3—C4—C5	−177.4 (3)
C17—O5—C18—C20	−131.2 (3)	C2—C3—C4—C5	0.3 (4)
C18—O5—C17—O4	−3.5 (4)	C3—C4—C5—C6	−5.1 (5)
C18—O5—C17—C1	175.9 (2)	C3—C4—C5—C7	173.4 (3)
C21—O7—C22—C23	−143.5 (3)	C4—C5—C6—C1	−21.6 (4)
C21—O7—C22—C24	95.8 (3)	C4—C5—C7—C8	−175.9 (3)
C22—O7—C21—O6	−8.4 (5)	C6—C5—C7—C8	2.6 (5)
C22—O7—C21—C1	169.7 (3)	C7—C5—C6—C1	160.0 (2)
C2—C1—C6—C5	51.1 (3)	C5—C7—C8—C9	177.4 (3)
C6—C1—C2—C3	−56.3 (3)	C7—C8—C9—C10	−4.1 (4)
C2—C1—C17—O4	−159.9 (2)	C7—C8—C9—C14	176.2 (3)
C2—C1—C17—O5	20.7 (3)	C8—C9—C10—C11	179.6 (2)
C17—C1—C2—C3	64.7 (2)	C8—C9—C14—C13	−179.8 (2)
C2—C1—C21—O6	126.9 (3)	C10—C9—C14—C13	0.5 (4)
C2—C1—C21—O7	−51.2 (3)	C14—C9—C10—C11	−0.7 (4)
C21—C1—C2—C3	−177.7 (2)	C9—C10—C11—C12	0.1 (4)
C6—C1—C17—O4	−38.2 (3)	C10—C11—C12—O2	−178.8 (3)
C6—C1—C17—O5	142.4 (2)	C10—C11—C12—C13	0.7 (4)
C17—C1—C6—C5	−71.6 (3)	O2—C12—C13—O3	−0.9 (4)
C6—C1—C21—O6	5.8 (4)	O2—C12—C13—C14	178.6 (2)
C6—C1—C21—O7	−172.3 (2)	C11—C12—C13—O3	179.6 (2)
C21—C1—C6—C5	171.6 (2)	C11—C12—C13—C14	−0.9 (4)
C17—C1—C21—O6	−112.3 (3)	O3—C13—C14—C9	179.7 (2)
C17—C1—C21—O7	69.6 (3)	C12—C13—C14—C9	0.3 (4)
